# A Word by Any Other Intonation: FMRI Evidence for Implicit Memory Traces for Pitch Contours of Spoken Words in Adult Brains

**DOI:** 10.1371/journal.pone.0082042

**Published:** 2013-12-31

**Authors:** Michael Inspector, David Manor, Noam Amir, Tamar Kushnir, Avi Karni

**Affiliations:** 1 Laboratory of Brain Imaging and Learning Research, University of Haifa, Haifa, Israel; 2 Department of Diagnostic Imaging, MRI Unit, the Chaim Sheba Medical Center, Tel Hashomer, Israel; 3 Department of Communication Disorders, Sackler Faculty of Medicine, Tel Aviv University, Tel Aviv, Israel; 4 MRI Unit, Rambam Medical Center, Haifa, Israel; University Of Cambridge, United Kingdom

## Abstract

**Objectives:**

Intonation may serve as a cue for facilitated recognition and processing of spoken words and it has been suggested that the pitch contour of spoken words is implicitly remembered. Thus, using the repetition suppression (RS) effect of BOLD-fMRI signals, we tested whether the same spoken words are differentially processed in language and auditory brain areas depending on whether or not they retain an arbitrary intonation pattern.

**Experimental design:**

Words were presented repeatedly in three blocks for passive and active listening tasks. There were three prosodic conditions in each of which a different set of words was used and specific task-irrelevant intonation changes were applied: (i) All words presented in a set flat monotonous pitch contour (ii) Each word had an arbitrary pitch contour that was set throughout the three repetitions. (iii) Each word had a different arbitrary pitch contour in each of its repetition.

**Principal findings:**

The repeated presentations of words with a set pitch contour, resulted in robust behavioral priming effects as well as in significant RS of the BOLD signals in primary auditory cortex (BA 41), temporal areas (BA 21 22) bilaterally and in Broca's area. However, changing the intonation of the same words on each successive repetition resulted in reduced behavioral priming and the abolition of RS effects.

**Conclusions:**

Intonation patterns are retained in memory even when the intonation is task-irrelevant. Implicit memory traces for the pitch contour of spoken words were reflected in facilitated neuronal processing in auditory and language associated areas. Thus, the results lend support for the notion that prosody and specifically pitch contour is strongly associated with the memory representation of spoken words.

## Introduction

Unlike tonal languages (e.g., Mandarin), in which tones (pitch) convey lexically meaningful information, the lexical identity of words does not usually change in non-tonal languages when the prosody of the word is changed. Prosody refers to acoustical patterns of speech units, and includes parameters such as the pitch contour of a speech unit, its volume and duration [1]. The pitch contour of a word, even in non-tonal languages, may convey important pragmatic information and can critically affect the way a given word is understood (e.g., indicate a question). It has been suggested that prosody may have a role in a word's recognition even in non-tonal languages [2]. For example, the relative pitch of an initial syllable may constrain the range of anticipated lexical candidates [2], [3]. Moreover, the complete prosodic pattern of a word may act as a framework to facilitate the retrieval of segmental phonology [4].

A plausible hypothesis would be that pitch contours (prosody) of spoken words would constitute an important perceptual attribute of the word as an auditory perceptual item. However, unlike other ‘incidental’ auditory input that may accompany the perception of a given spoken word (environmental noise, speaker's gender or age) that may perhaps be ignored in order to better extract the lexical-semantic input (possibly by a process of ‘perceptual normalization’ [5]–[11]), the prosody and specifically a perceptual feature such as the pitch contour of a spoken word, should not be ignored as it can facilitate its disambiguation. Church & Schacter [9] have previously shown that minimal changes in the pitch contour of word stems and changes in the intonation of repeated words were associated with an increased reaction time (decreased repetition priming) in word recognition tasks, and concluded that the pitch contours of spoken words are implicitly remembered. Thus, changing the pitch contour may have a different effect on word processing compared to, for example, changing a speaker voice. A recent fMRI study [12] found, in line with the results of an earlier PET brain imaging study [13], that the same word when reproduced by a different person was not recognized, both behaviorally and in terms of the brain imaging measures, as a novel stimulus.

Here we used fMRI to investigate the role of prosody in the representation of single spoken words by addressing the question of whether a change in the prosody, specifically, the pitch contour (intonation) which was associated with a previously heard spoken word, may result in a change in the ability of auditory and language brain systems to process it as the same lexical entity when it is heard again. The current study was motivated by the consideration that because different intonations may facilitate or inhibit word recognition and may potentially entail different interpretations of the utterance, differences in intonation should not be automatically ignored or masked by early processing stages. Thus, the general conjecture was that the pitch contour of a previously heard spoken word may be implicitly remembered and if this feature is changed, the processing of the (same) target word would be relatively impeded when reencountered.

When a stimulus of a given modality is perceptually identical to one encountered beforehand, or is closely related to it, the ability to process the stimulus upon repetition is often enhanced relative to its processing when encountered for the first time, irrespective of intentional recollection of the previous encounter, a phenomenon referred to as repetition priming (RP) [14]. RP presumably reflects implicit memory, though not necessarily long-term memory [15] and is expressed behaviorally by reduction in response latency and by improvement in accuracy of response to the stimulus. There is good support, mainly from studies of the visual system, for the notion that repetition suppression (RS), a decrease in electrophysiological and metabolic brain responses to repeated stimuli, is a counterpart of RP in brain imaging measures [16], [17]. We tested whether RS could be demonstrated in auditory and language processing brain areas during repeated auditory presentations of words and specifically, whether RS would be reduced or abolished during repeated auditory presentations of the same words but with a changed pitch contour in each successive presentation.

There are substantial behavioral data indicating the existence of RP for words presented in the auditory modality [10], [11], [18]. Moreover, in analogy to the results reported in priming studies of words in the visual modality [19] diminished auditory priming was demonstrated when changes in acoustical parameters were introduced between prime and test presentations of the same words [10], [11], [18]. In the visual modality, changing specific visual features of words (fonts, letter case) between the initial presentation and the test, significantly reduced the RS of the BOLD signals in visual processing areas (e.g., in a semantic categorization task ([20].The main hypothesis in the current study was that pitch contour modulations of repeated auditory words would reduce RS in auditory and in language processing brain areas.

To test this hypothesis, we first had to establish whether RS could be consistently evoked in auditory and language processing areas in response to repeated auditory presentations of words. Two PET studies and five fMRI studies, have specifically addressed the phenomenon of RS during repeated auditory stimulation, one study using environmental sounds and the other five using words or sentences [12], [13], [22], [23], [24], [25]. In only three of these studies (all three studies using fMRI) significant RS was showed: in the right superior temporal gyrus in the earliest one [21], in the right STG and left posterior MTG/STG and temporal peri-sylvian language processing areas in a latter study [23] and in the right middle/posterior STS and right associative auditory cortex, in response to spoken words in an acoustically degraded format, in a more recent one [24]. RS like effects, non-significant statistically, were reported in the auditory cortex [22]. Most studies [12], [13], [22], [25] however, failed to show significant RS in modality specific auditory processing areas, although significant RS was found in visual, frontal (including the left IFG) and multimodal processing areas. To increase the likelihood of obtaining RP and PS effects in the auditory modality, we used, in the current study, a semantic categorization task. Behaviorally, semantic categorization judgment tasks on words were found to evoke significant RP effects in both the visual and the auditory modalities [26], [27], [28], [29]. A fMRI study using a semantic categorization task [27] demonstrated significant RS effects in the left prefrontal cortex. Auditory semantic categorization judgment tasks were found to activate the left temporal regions (superior and middle temporal gyri) as well as the inferior frontal regions and anterior prefrontal regions [26].

We show that in semantic categorization tasks, as well as in a non-semantic listening task, significant RS occurred in the primary auditory cortex, bilaterally, and bilaterally in superior and middle temporal gyri and the superior temporal sulcus (BA 21 22), as well as in the inferior frontal gyri, for repeated auditory presentations of words in a flattened monotonous pitch (modulation M). Robust RS was found also for repeated words with complexly modulated pitch contours, provided these remained unchanged across successive presentations (modulation P) in both auditory and temporal language areas. However, when the repeated words' pitch contour was changed between successive repetitions (modulation V), the RS effect was significantly diminished (i.e. was eliminated) in these areas.

## Methods

### Participants

Eleven (7 women and 4 men) right-handed university students (age, 22–28 years) were studied. Right handedness was established using the Edinburgh handedness inventory [30]. All participants spoke Hebrew as their native language. None of the participants had reported a neurological or psychiatric illness or a history of language or communication disorder, and none used medications on a regular basis. Participants gave written informed consent. The study was approved by the ethics committee of the Chaim Sheba Medical Center.

### Stimuli

The auditory stimuli were pre-recorded and pitch-contour manipulated Hebrew words. The words were nouns signifying places in urban or non-urban environments in the first semantic categorization task, and nouns signifying either items of apparel (clothing and jewelry) or non-apparel in the second semantic categorization task. Nouns signifying vehicles were used in the non-semantic listening task as well as in a semantic categorization task which was used for defining the brain regions of interest (ROIs). The mean duration of the words in all tasks was 892.2 milliseconds (msec) (SD = 149).

A single male speaker was recorded for all stimuli, using Goldwave 5.08 software, a Sound Blaster Audigy 2 NX USB sound card and a directional microphone. The sampling rate was 44.1 kHz. The prosodic manipulations constituted changing only the pitch contour, without affecting syllable intensity or duration. These were carried out using the Praat software package [31], which provides convenient means to specify a modified F0 contour as a piecewise-linear curve, and then re-synthesize the speech to fit the curve. d'Allesandro's perceptual criteria [32] were applied to each glissando in the F0 contour separately. These criteria enabled the generation of a library of distinct pitch templates for each word, based on a quantitative, objective measure. Only manipulations with glissandi which were measured to be above the glissando threshold according to d'Allesandro's perceptual criteria [32] and which were judged perceptually highly distinct and intelligible by two independent Hebrew native speakers, were used.

**Figure 1:**
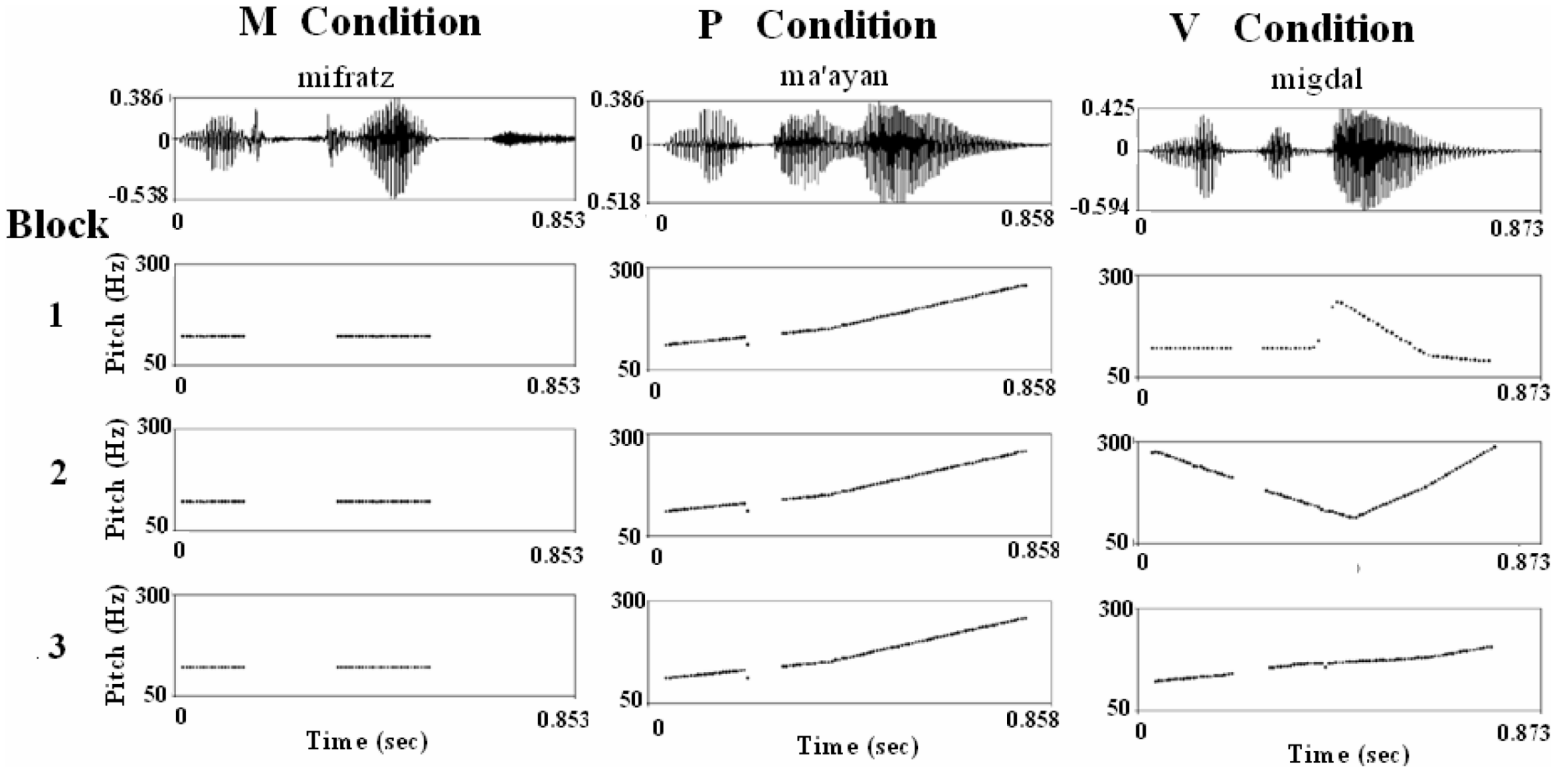
The pitch contours of three different words, as examples for each of the different prosodic conditions: M, P or V. The word “mifratz” (a bay) is shown as an example for a repeated monotonous pitch contour (M condition blocks). The word “ma'ayan” (a spring) is shown with a consistent pitch modulation across its three repetitions (P condition blocks) and the word “migdal” (a tower) is shown in three different pitch contours (V condition blocks).

To reduce extraneous priming effects, words with a different number of syllables were used in the two categorization tasks (2 syllables and 3 syllables, in the urban – non-urban and the apparel – non-apparel tasks, respectively). Words had CV syllabic structure (i.e., a consonant followed by a vowel, that is, CV-CV for the two syllable words and CV-CV-CV for the three syllable words) or contained also CVC syllabic structure (CV-CVC for two syllable words and CV-CV-CVC or CVC-CV-CV for the three syllabic words). The pitch contour manipulation was based upon changing F0 frequencies at specific anchor points. For each target word in the apparel – non-apparel task, 3 anchor points were determined (the initiation of voicing, the middle of the second vowel and voicing termination) and one out of four different frequency values was selected for each point: 80, 120, 180 or 220 Hertz (Hz) (Fig. 1). Thus, the musical intervals between two adjacent points were either 7 semitones (a “fifth”; as between 80 Hz and 120 Hz) 14 semitones (as between 80 Hz and 180 Hz), 3.47 semitones (as between 180–220 Hz), 10.47 semitones (as between 120–220 Hz), 17.5 semitones (as between 80 Hz and 220 Hz) or zero if two adjacent points were of the same frequency. In a similar manner, in the urban – non-urban categorization task, 3 anchor points were determined for each target word (the initiation of voicing, the beginning of the second vowel and voicing termination). The F0 frequencies of the anchor points were 87–286 Hz with the constraint that the pitch contour of the whole word was distinct according to d'Allesandro's perceptual criteria [32].

The mean lexical frequency of the words, in the different semantic tasks, was relatively low (9.41±6.44; 3.38±2.33; 3.8333±1.472, mean ± SD per million, urban-non-urban, apparel- non-apparel, vehicle tasks, respectively) (Word frequency index of Hebrew words, R. Frost, Hebrew University, Jerusalem).

### Behavioral tasks and set-up

A short practice on a semantic categorization task (whether the target word related to urban or non-urban environments), which included auditory presentation of six nouns, each with a distinct pitch contour, was provided for all participants, shortly before being placed in the magnet. The words used in practice were not repeated in the actual experiment.

**Figure 2:**
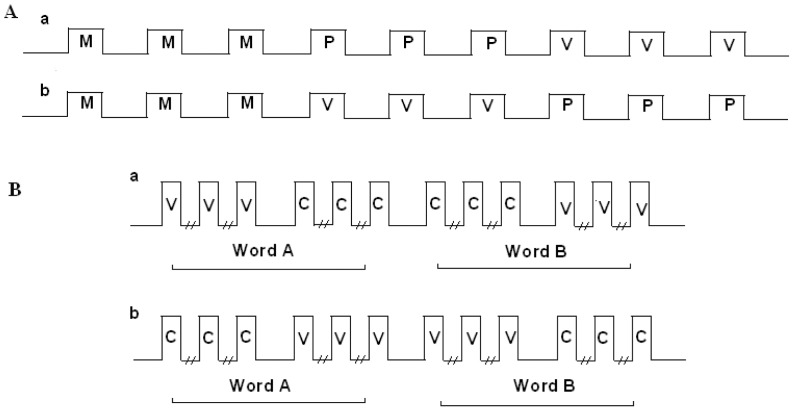
The block/mini-block sequences used in (A) the semantic categorization tasks and (B) the non-semantic task. (A) Two block sequences (a, b) were counterbalanced for the two semantic categorization tasks, and between participants. Each semantic task consisted of 3 sets of 3 repeating blocks. Each set included its unique list of words in one of three prosodic modulations: M- monotonic modulation, P- persistent prosodic modulation or V- variably changing modulation. The durations of each task block and rest interval were 15 and 18 seconds, respectively. (B) For the non-semantic task each word (word A, word B) was presented in two sets of three mini-blocks in one of two prosodic modulations: C - Consistent pitch contour in all repetitions of the same word; or V – variable, changing pitch contour between repetitions of the same word Two mini-block sequences (a, b) were counterbalanced between the participants.. Each mini-block's duration was 6 sec and the duration of the between mini-blocks intervals was 9 sec; intervals between sets of mini-blocks −18 sec.

In the scanner, participants performed the tasks in a fixed order, but the order of conditions, within tasks, was pseudo-randomized and counterbalanced (Fig. 2). Target words were presented in the auditory modality and in a blocked design. Participants had a maximal interval of 2000 milliseconds to respond to each target word. First, categorization of nouns signifying vehicles as traveling by land or traveling by air or water (vehicle task) was used to map functional regions of interest (ROIs) (42 scans). Second, two semantic categorization tasks and a non-semantic listening task were used to study RS effects and their modulation by pitch contour changes; first semantic task: categorization of nouns as urban – non-urban (108 scans); second semantic task: categorization of nouns as apparel – non-apparel (108 scans). These were followed by the non-semantic listening task, in which participants listened to two repeating words and were required to press a response button at the end of each auditory presentation of a word (92 scans). Finally, passive listening to iterated rippled noise [33], [34] was used to functionally define the primary auditory cortex (A1) (64 scans). Each participant completed all of the above tasks.

In the MRI scanner, the auditory stimuli were presented to the participants using a MR-compatible Audio system (Avotec, USA). Auditory stimuli were presented using on the background noise of the MRI scans. To ensure that the stimuli were heard with sufficient loudness and clarity, words which were not part of the lists used in the experiment, presented with scanner noise, served to adjust the presentation volume at the beginning of each scanning session. Participants responded by pressing one of two response buttons with their right hand (index and middle finger) using the Lumina response box (Cedrus Corporation, CA, USA). Reaction times (RT) and accuracy of the response for each target word was recorded for off-line analysis. RT was defined from the start of the stimulus. The mean duration of the words in all tasks was 892.2 milliseconds (msec) (SD = 149). The mean duration of the words in all tasks was 892.2 milliseconds (msec) (SD = 149). The mean duration of the tri-syllabic and di-syllabic stimuli were 949.75 msec (SD = 137.4) and 790.9 msec (SD = 71.9), respectively. In all of the above tasks participants were instructed to maintain gaze fixation on a white circle (0.4°) at the center of a black background which was back-projected on a screen and viewed through a mirror device. The stimuli presentations and response recordings were implemented using Cogent2000 (http://www.vislab.ucl.ac.uk/Cogent2000).

### Prosodic modulations (conditions)

The semantic categorization tasks consisted of sets of three blocks of task performance and each block was preceded and followed by rest intervals wherein no auditory stimuli were presented (Fig. 2 A). Within each set, each of the three task blocks consisted of 6 target words with the order of the words changed across blocks in a pseudo-random manner. The vehicle categorization task consisted of a single set of three task blocks with all of the words presented in a fixed flattened monotonous pitch contour (122 Hz, the mean pitch of the speaker's voice).

The urban – non-urban and the apparel – non-apparel categorization tasks consisted of three sets each, corresponding to three conditions: monotonous modulation (M) - in which a single fixed flattened monotonous F0 of 122 Hz was applied to all of the target words in the three successive blocks of the set (Figure 1) (i.e., a single pitch contour in the three blocks); persistent prosodic modulation (P) - in which a unique pitch contour was assigned to each target word and consistently maintained across its three presentations in the three successive blocks of the set (Figure 1) (i.e., a total of 6 different pitch contours in the three blocks, one per each word); variable, changing, modulation (V) - in which the pitch contour of each target word was different in each of its three presentations across the successive blocks of the set (Figure 1) (i.e., a total of 18 different pitch contours in the three blocks). The sets were presented in two different sequences M-P-V and M-V-P counterbalanced between the two semantic categorization tasks and across participants (Fig. 2 A). A different set of six words was used in each of the three prosodic conditions (M, P or V) in each of the two semantic categorization tasks (a total of 36 words). In the non-semantic task, a single target word was presented in the three successive mini-blocks within each set, with six successive presentations of the word in each mini-block (Fig. 2 B). Two target words were used. Two conditions were tested for each word. In one condition (C condition), the target word was presented 18 times in the set with a fixed pitch contour (for one of the target words in a rising and in the other a falling pitch contour). In the other condition (V condition), each target word was presented with a continuously changing pitch contour, thus presenting 18 different unique intonations of the same word in the set. No semantic decision had to be taken concerning these words, but to maintain vigilance, the participants were asked to press a button at the moment the auditory presentation of each word ended. The order of the words and conditions were counterbalanced across participants.

### Functional MRI procedure

Functional magnetic resonance imaging (fMRI) was conducted at the Chaim Sheba Medical Center, Tel Hashomer, on a 3T (GE, Signa) whole body MRI high definition (HD) system equipped with a birdcage head coil. Structural anatomical images were obtained using 3D IR prepared FSPGR T_1_ weighted scans with a resolution of 1 mm^3^. The functional imaging sequence (BOLD contrast) was gradient-echo EPI with the following parameters: repetition time (TR) = 3000 msec, echo time (TE) = 30 msec, flip angle (FA) 90°. 36 contiguous axial slices (slice thickness = 3 mm with gap of 0.4 mm, FOV = 220×220 mm; 64×64 within slice resolution) parallel to the AP-PC plane were obtained with full coverage of the cerebral hemispheres and cerebellum.

### Behavioral Data analysis

The participants' responses were recorded during the functional MRI sessions and reaction times (RT) and response accuracy were computed. Repeated measure ANOVAs were run to compare RTs for correct responses across the repeated blocks (3 repeating blocks in each prosodic modulation condition) and the different stimulus conditions (prosodic modulations: M, P, V) in each semantic categorization task. The data obtained from the two semantic categorization tasks were analyzed together.

### fMRI data analysis

The imaging data were analyzed using SPM2 (Wellcome Department of Cognitive Neurology, London, U.K). The first four volumes were discarded from each session to allow for T_1_ equilibration effects. Following image reconstruction and motion correction, all images were smoothed using a 6 mm FWHM Gaussian kernel. Global scaling was not performed. Each task (set) was modeled separately although the two semantic categorization tasks were also analyzed together. A boxcar function convolved with canonical hemodynamic response function (HRF) with derivation was applied for each subject. Contrasts of parameter estimates were used to generate statistical maps (SPMs) of the t-statistic for each experimental set.

ROIs were defined using MarsBaR version 0.35 (MARSeille Boîte À Région d'Intérêt) [35]. Individual functional ROIs were based on BOLD activity during the performance of the vehicle task and passive listening to iterated rippled noise was used to define A1. The functional ROIs derived from these tasks, were then combined with anatomic ROIs using Pick-Atlas (v.2; FMRI Laboratory, the Wake Forest University School of Medicine) and generated ROI masks based on the Talairch Daemon database. The anatomic ROIs used were: Brodman areas (BA) 21 &22 (middle and superior temporal gyrus- MTG and STG), BA 44 &45 (Inferior frontal gyrus-IFG), BA 41 (primary auditory cortex – A1) and supplementary motor area (SMA). Using MarsBar, the model design of each set (task) was imported and t contrasts for the voxels within each individual functional ROI were extracted. A new summary time course for each ROI was derived, representing a mean of all the voxel values within the ROI for each time point and yielding t statistics. The analysis also yielded contrast values (CVs), i.e. the effect size for the t statistic that SPM stores for each voxel in the images series. The contrast values were then used for second level analysis by General Linear Model procedure for repeated measures with Bonferroni tests for unplanned multiple paired comparisons. For the use of contrast values (effect sizes for the t statistics) extracted by MarsBar for secondary statistical analysis, see: [36]–[38]. Whenever the BOLD signals in a given ROI were negative, the data were not included in the analysis.

To assess the effects of repetition (3 repeating blocks) in the three stimulus conditions (prosodic modulations: M, P, V) on the behavioral (RT measurements) and separately on the BOLD contrast values, repeated measures ANOVAs were used, in a mixed design, with the task as a between-observations factor (two observations per participant). Bonferroni correction for multiple comparisons, was used in the post hoc comparisons between pairs of blocks (the criterion for significance set at p<0.05/repetitions; i.e., *p*<0.016).

## Results

### Semantic categorization tasks: Behavioral data

Behavioral data were available for 10/11 participants (technical failure in one participant). High accuracy was attained in all three conditions tested. The mean percentage of correct responses across both semantic tasks was 98.8%, 98.6% and 97.5% in the M, P and V modulations, respectively. RTs of correct responses were analyzed for repetition effects.

**Figure 3:**
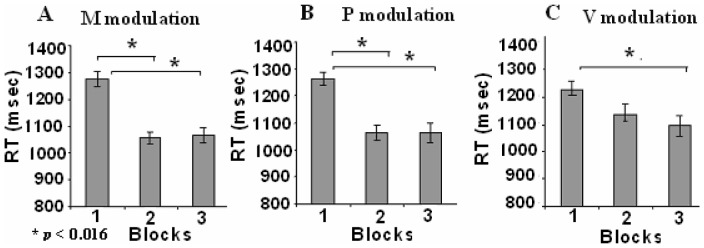
Mean reaction Times (RT) and SEM in the two semantic categorization tasks (combined). The three prosodic modulations: (**A**) M -monotonic modulation; (**B**) P -persistent prosodic modulation; (**C**) V- variably changing modulation. (*p<0.016, by Bonferroni test for multiple comparisons).

A repeated measures ANOVA showed no significant interaction between the two tasks and repetitions {*F* (2, 36) = 1.48, *p* = 0.241}. However, there was a significant main effect of repetition (i.e., RP) {*F*(2, 36) = 44.35, *p*<0.001} and a significant interaction of repetition and stimulus conditions {*F*(4,72) = 3.54, *p* = 0.011} (Fig. 3 A–C) indicating that the repetition effects were of a significantly different magnitude in the three prosodic modulation conditions.

A further repeated measures ANOVA was used to asses RP effects in each stimulus condition separately. In the M modulation condition, a significant difference in RT was found between blocks {*F*(2,36) = 42.09, *p*<0.001}. Pair-wise comparisons showed a significant decrease in RT between blocks 1 and 2 (mean difference (MD) = 219.01 msec, standard error (SE) = 30.28, *p*<0.001) and between blocks 1 and 3 (MD = 211.43 msec, SE = 29.42, *p*<0.001) but not between blocks 2 and 3 (MD = 7.57 msec, SE = 20.49, *p* = 1) (Fig. 3A). There was a significant difference in RT between blocks also in the P modulation condition {*F*(2, 36) = 26.82, *p*<0.001}. Pair-wise comparisons showed a significant decrease in RT between blocks 1 and 2, (MD = 197.66 msec, SE = 31.42, *p*<0.001) and between blocks 1 and 3 (MD = 197.59 msec, SE = 33.57, *p*<0.001) but not between blocks 2 and 3 (MD = 0.07 msec, SE = 33.57, *p* = 1) (Fig. 3B). In the V modulation, the repetition effects although significant {*F*(2, 36) = 8.85, *p* = 0.001}, were significantly smaller than the corresponding ones in the M and P conditions (as indicated by the significant interaction of repetition and stimulus condition in the initial ANOVA). Pair-wise comparisons indicated (Fig. 3C) that the decrease in RT between block 1 and 2 and between blocks 2 and 3 were not significant (MD = 86.22 msec, SE = 36.67, *p* = 0.075; MD = 44.38, SE = 29.57 msec, *p* = 1, respectively). Only the decrease in RT between blocks 1 and 3 was significant (MD = 134.01 msec, SE = 30.63, *p* = 0.001) with the absolute decrease in RT significantly smaller than that achieved in the corresponding M {*t*(19) = 2.47, p = 0.023} but not in comparison with the P condition {*t*(19) = 1.77, p = 0.093}. The absolute decrease in RT between blocks 1 and 2 in the V condition was significantly smaller than that achieved in the corresponding M and P conditions {*t*(19) = 4.15, *p* = 0.01; *t*(19) = 2.56, *p* = 0.019; respectively}.

Taken together, our results showed that in the V modulation condition, in which the words' intonation was changed between each successive repetition, the RP effects, between blocks 1 and 2, were not statistically significant, in contrast to the clear RP effects in both the M and P conditions.

### Semantic categorization tasks: fMRI data

**Figure 4:**
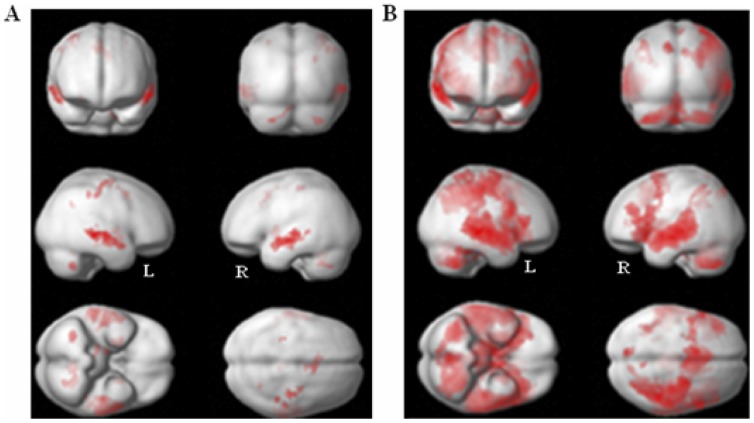
Regions of activation induced by the semantic categorization tasks in all three prosodic modulations. (**A**) High threshold Map: p<0.05; FWE corrected, Extent threshold: k = 27 voxels. (**B**) Low threshold Map: p<0.001, non -corrected; Extent threshold: k = 27 voxels.

Due to head movement artifacts the fMRI data of one participant were omitted from the analysis. The brain areas in which significant activation was evoked by the performance of the semantic categorization tasks are listed in Table 1 and shown in Fig. 4. Each of these significantly activated regions, in each hemisphere (except for the SMA region) was tested for activation in each participant and in each block separately.

**Table 1:** Regions of activation in both semantic categorization tasks in all three prosodic modulation conditions (center of clusters, extent threshold: k = 27 voxels).

*Talairach Coordinates*						
*Regions*	*BA*	*X*	*Y*	*Z*	*Z-score*	*voxels*
L. MT/ST	21 22	60	−30	−4	7.32	947
L, R. SMA & Cingulum	6	8	10	46	7.08	231
R. MT/ST	21 22	−58	−2	−12	6.61	887
L. Pre/Post central gyrus	4	36	−22	50	6.38	137
L. Parahippocampal gyrus	35	16	−26	−16	6.25	66
L. Parietal inf., SMG	40	44	−38	48	6.13	43
L. Putamen, Pallidum		24	−2	−2	5.97	204
L. Cerebellum		34	−58	−42	5.86	122
R. Cerebellum		−26	−68	−44	5.77	208
R. Primary motor cortex		−30	−26	52	5.36	29
L. Primary auditory cortex	41	44	−26	6	5.21	298
		58	−18	12	4.52	
		46	−34	14	4.49	
R. Primary auditory cortex (A1)	41	−58	−22	6	4.48	125
		−48	−28	6	4.44	
		−54	−32	14	4.14	
L. IFG	44/45	52	28	6	5.27	378
		36	22	4	4.99	
		52	10	22	4.83	
R.IFG	44/45	−50	30	8	5.14	420
		−48	16	16	4.79	
		−48	16	4	4.12	

Center of cluster given in Talariach coordinates. MT/ST = middle temporal/superior temporal lobes (encompassing both banks of the superior temporal sulcus); SMA = supplementary motor area; SMG = supramarginal gyrus; IFG = inferior frontal gyrus. Some brain areas showed more than one center of activation. For all regions except left and right IFG: p<0.05, FWE correction. For left and right IFG: p<0.001.

#### Left and right STG-MTG

The STG and MTG (STG-MTG) were treated as a single ROI encompassing both banks of the STS. A significant and strong correlation was detected between the RTs pooled across all blocks and all prosodic modulations and the BOLD contrast values (CV) in the left and the right (Pearson, r = 0.448, *p*<0.001; r = 0.504, p<0.001, N = 138; respectively).

A separate repeated measures ANOVA was used on the CVs from each hemisphere, to assess the effects of repetition and the different stimulus conditions, across both tasks. No significant interactions were detected between tasks and repetitions {*F*(2, 36) = 1.088, *p* = 0.348; *F*(2, 36) = 0.78, *p* = 0.46; left and right STG-MTG respectively}. However, there was a significant interaction between stimulus conditions and repetitions in both hemispheres {*F*(4,72) = 2.56, *p* = 0.045; *F*(4,72) = 3.18, *p* = 0.018, respectively} indicating different repetition effects in the different prosodic modulation conditions.

Three repeated measure ANOVAs on the 3 blocks of each stimulus condition, separately, across both tasks, showed a significant difference between repeating blocks (i.e., RS) in the M modulation {*F*(2, 36) = 16.3, *p*<0.001; *F*(2,36) = 27.93, *p*<0.001; left and right STG-MTG, respectively] and in the P modulation {*F*(2,36) = 8.27, p = 0.001; *F*(2,36) = 5.82, *p* = 0.001} but not in the V modulation {*F*(2,36) = 1.28, p = 0.29; F(2,36) = 2.149, *p* = 0.131}.

**Figure 5:**
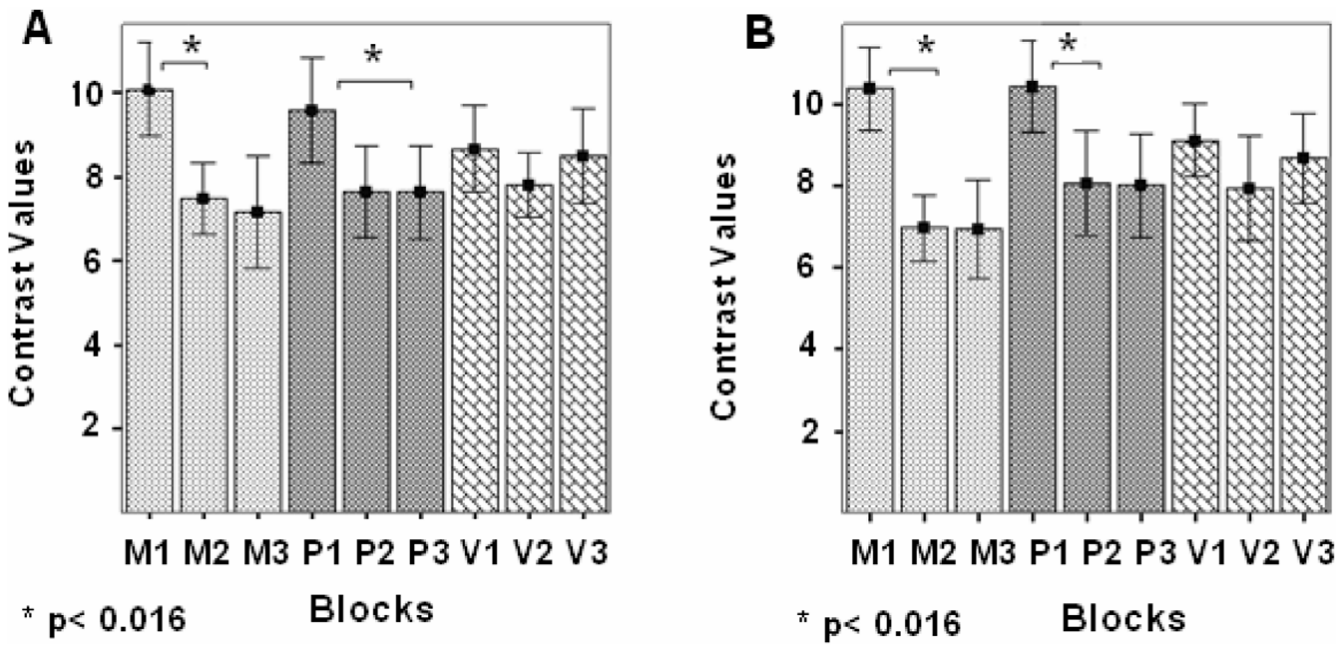
ROI analysis in STG-MTG in the two semantic categorization tasks, pooled together. Contrast values (CV) and SEM in the three repeating blocks of each of the three prosodic modulations (M, P, and V). (**A**) Left STG- MTG; (**B**) Right STG-MTG. Repetition suppression (RS) effects were found in the M and P modulations (*p<0.016, Bonferroni test for multiple comparisons).

Post-hoc pair-wise comparisons showed significant decreases of the CV (i.e., significant RS) in the left STG-MTG between block 1 and 2 in the M modulation (MD = 2.59, SE = 0.527, *p*<0.001) (Fig. 5 A). In the right STG-MTG significant RS was found between blocks 1 and 2 in the M and P modulations (MD = 3.39, SE = 0.52, *p*<0.001; MD = 2.35, SE = 0.7, *p* = 0.01, respectively) (Fig. 5 B). Significant RS was detected also between blocks 1 and 3 in the P modulation (MD = 1.93, SE = 0.516, *p* = 0.004; MD = 2.43, SE = 0.54, *p* = 0.001; left and right STG-MTG, respectively). However, no significant RS was detected in the V modulation between any pair of repeating blocks.

#### Left and right A1

A repeated measures ANOVA on the CVs (with data from 7 participants in each task for the right A1 and data from 7 and 9 participants from the two tasks for the left A1, i.e. 16 observations for the left A1 and 14 observations for the right A1) showed no significant interactions between tasks and repetitions in either A1 {*F*(2, 28) = 0.16, *p* = 1.64, *F*(2, 24) = 0.52, *p* = 0.59, left and right A1 respectively}. A significant interaction between stimulus condition (different prosodic modulation) and repetition was found in the right A1 {*F*(4, 48) = 3.28, *p* = 0.018} but not in left A1 {*F*(4, 56) = 1. 64, *p* = 0.212}.

Three repeated measure ANOVAs on the 3 blocks in each stimulus condition, separately, showed a significant difference between repeating blocks (i.e., RS) in the left and right A1 in the M modulation {*F*(2,36) = 11.05, *p*<0.001; *F*(2,36) = 8.29, *p* = 0.001, respectively} and in the right A1 but not left A1 in the P modulation {*F*(2,28) = 8.06, p = 0.002; *F*(2,28) = 3.13 *p* = 0.059, respectively} and no significant RS in the V modulation {*F*(2,32) = 0.82, *p* = 0.92; F(2,26) = 0.48, *p* = 0.62, left and right A1, respectively}.

**Figure 6:**
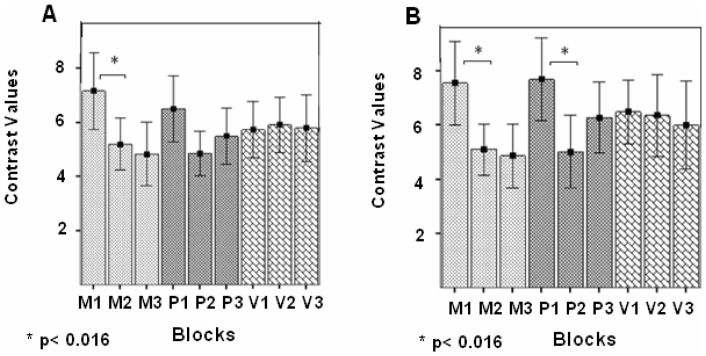
ROI analysis in A1 in the two semantic categorization tasks, pooled together. Contrast values (CV) and SEM in the three repeating blocks of each of the three prosodic modulations (M, P, and V). (**A**) Left A1; (**B**) Right A1. Repetition suppression (RS) effects were found in the M and P modulations in right A1 and in the M modulation in left A1 (*p<0.016, Bonferroni test for multiple comparisons).

Post-hoc pair-wise comparisons showed significant RS effects in the left A1 between block 1 and 2 in the M modulation (MD = 1.97, SE = 0.55, *p* = 0.007) (Fig. 6 A) and in the right A1 between block 1 and 2 in the P modulation (MD = 2.725, SE = 0.717, *p* = 0.007) (Fig. 6 B). Also, in the M modulation, significant RS was found in A1, bilaterally, between block 1 and 3 (MD = 2.33, SE = 0.63, p = 0.005, MD = 2.66; SE = 0.81, *p* = 0.015, left and right A1 respectively). There was no statistically significant RS in A1, in either hemisphere, in the V modulation between any pair of repeating blocks (*p* = 1 for all comparisons).

#### Left and right inferior frontal gyrus (Brodman 44/45)

A repeated measures ANOVA was used to assess the effects of the different stimulus conditions and repetitions on the CVs in the two tasks pooled together (with data from 7 participants on the urban/non-urban for IFG bilaterally, and of 9 and 8 participants on the apparel–non-apparel task for left and right IFG respectively, i.e. 16 observations for the left A1 and 15 observations for the right A1). No significant interactions between the tasks and the repetitions were found in either IFG {*F*(2, 28) = 1.22, p = 0.3, *F*(2,26) = 1.51, p = 0.24, left and right IFG, respectively}. A significant interaction between stimulus conditions and repetition was found in both the left and right IFG {*F*(4, 56) = 2.74, *p* = 0.037; *F*(4, 52) = 2.68, *p* = 0.041, respectively}.

Repeated measures ANOVAs on each stimulus condition separately, showed a significant difference between repeating blocks (i.e., RS) in the M modulation }*F*(2, 32) = 8.69, p = 0.01; *F*(2, 30) = 5.41, *p* = 0.01, left and right IFG, respectively{ but not in the P {*F*(2, 30) = 5.5, *p* = 0.09; *F*(2, 26) = 1.3, *p* = 0.28, left and right IFG, respectively} or V {F (2, 30) = 0.24, *p* = 0.78; *F*(2, 28) = 1.5, *p* = 0.34, left and right IFG, respectively} modulations.

**Figure 7:**
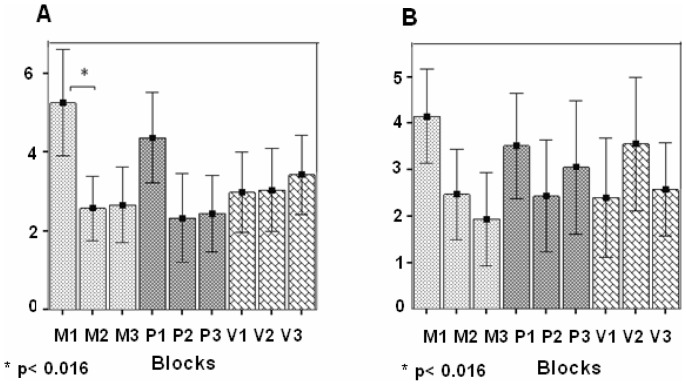
ROI analysis in IFG in the two semantic categorization tasks, pooled together. Contrast values (CV) and SEM in the three repeating blocks of each of the three prosodic modulations (M, P, and V). (**A**) Left IFG; (**B**) Right IFG. Repetition suppression (RS) effects were found in the M modulation in left IFG (*p<0.016, Bonferroni test for multiple comparisons).

Post-hoc pair-wise comparisons showed significant RS in the left IFG in the M modulation, between block 1 and 2 (MD = 2.6, SE = 0.66, *p* = 0.03) (Fig. 7 A) and between block 1 and 3 (MD = 2.58, SE = 0.88, *p* = 0.03). No significant RS was detected by the post-hoc comparisons in the left IFG between block 1 and 2 or between block 1 and 3, in the P and V modulations, or in right IFG in the M modulation (Fig. 7 A, Fig. 7 B).

### Non-semantic task: Behavioral data

A repeated measure ANOVA was used to assess the effects of repetition (3 mini blocks) in the two prosodic modulation conditions: constant pitch modulation (C modulation) and variable pitch (V modulation). There was a significant difference in the mean RT between the different prosodic modulation conditions {*F*(1,9) = 7.12, *p* = 0.026} (mean RT 736 msec (SE = 14.8) and 709 msec (SE = 11.2) in the V and C modulation conditions, respectively).

No significant interaction was found between the prosodic modulation conditions and repetition {*F*(2,18) = 2.06, *p* = 0.15}. No significant RP was found across repeating mini-blocks in the two prosodic modulations pooled together {*F*(2,18) = 1.66, *p* = 0.21}. In pair-wise comparisons, no statistically significant difference was found between any pair of blocks in either prosodic modulations.

### Non-semantic task: fMRI data

The brain areas in which significant activation was evoked by task performance in the non-semantic task were the STG-MTG and A1, bilaterally. These areas were further analyzed as separate ROIs.

#### Left and right STG-MTG

A repeated measure ANOVA was used to assess the effects of repetition (3 mini-blocks) in the two stimulus conditions (C, V). A significant interaction between stimulus conditions and repetitions was found in the left STG-MTG {*F*(2,18) = 4.09, *p* = 0.034}, but not in the right STG-MTG {*F*(2,18) = 2.85, *p* = 0.084}.

A repeated measure ANOVA on each stimulus condition, separately, showed a significant difference in CVs between the mini-blocks (i.e., RS) in the C modulation condition {*F*(2,18) = 15.43, *p*<0.001; *F*(2,18) = 16.2, *p*<0.001, left and right STG-MTG, respectively}, but not in the V prosodic modulation condition {*F*(2,18) = 0.84, *p* = 0.44; *F*(2,18) = 0.84, *p* = 0.44, left and right STG-MTG, respectively}.

**Figure 8:**
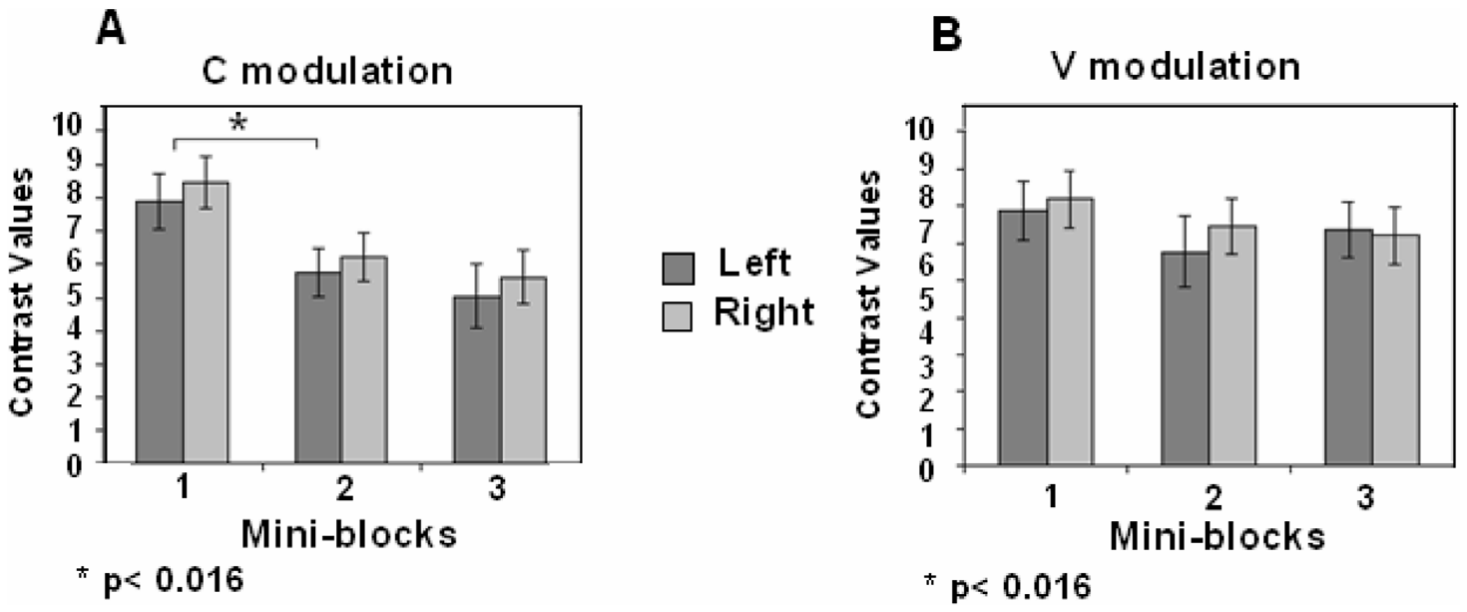
ROI analysis, left and right STG-MTG in the three repeating mini-blocks of the non-semantic task. Contrast values (CV) and SEM are shown. (A) During C modulation - constant pitch contour for all repetitions of the same word; (B) during V modulation - variable pitch contour between repetitions of the same word. Repetition suppression (RS) effect was found only in the C modulation (*p<0.016, Bonferroni test for multiple comparisons.

Pair-wise comparisons showed significant RS in the C prosodic modulation between mini-blocks 1 and 2 (MD = 2.13, SE = 0.44, *p* = 0.003; MD = 2.27, SE = 0.41, *p* = 0.001, left and right STG- MTG, respectively) (Fig. 8 A). No significant RS was detected in the V modulation condition between any pair of mini-blocks (Fig. 8 B).

#### Left and right A1

A repeated measure ANOVA showed a significant interaction between the two prosodic modulations and mini blocks in the left A1 {*F*(2,16) = 3.9, *p* = 0.042}, but not in right A1 {*F*(2,18) = 0.33, *p* = 0.72}. Thus, the repetition effects were significantly different only in the left A1 in the two prosodic modulation conditions.

A repeated measure ANOVA on each stimulus condition, separately, showed a significant difference between the mini-blocks in the C modulation condition (i.e., RS) {*F*(2,18) = 14.307, *p*<0.001; *F*(2,18) = 3.885, *p* = 0.04, left and right A1 respectively}, but not in the V modulation condition {*F*(2,16) = 2.463, *p* = 0.112; *F*(2,18) = 0.171, *p* = 0.844, left and right A1 respectively}.

**Figure 9:**
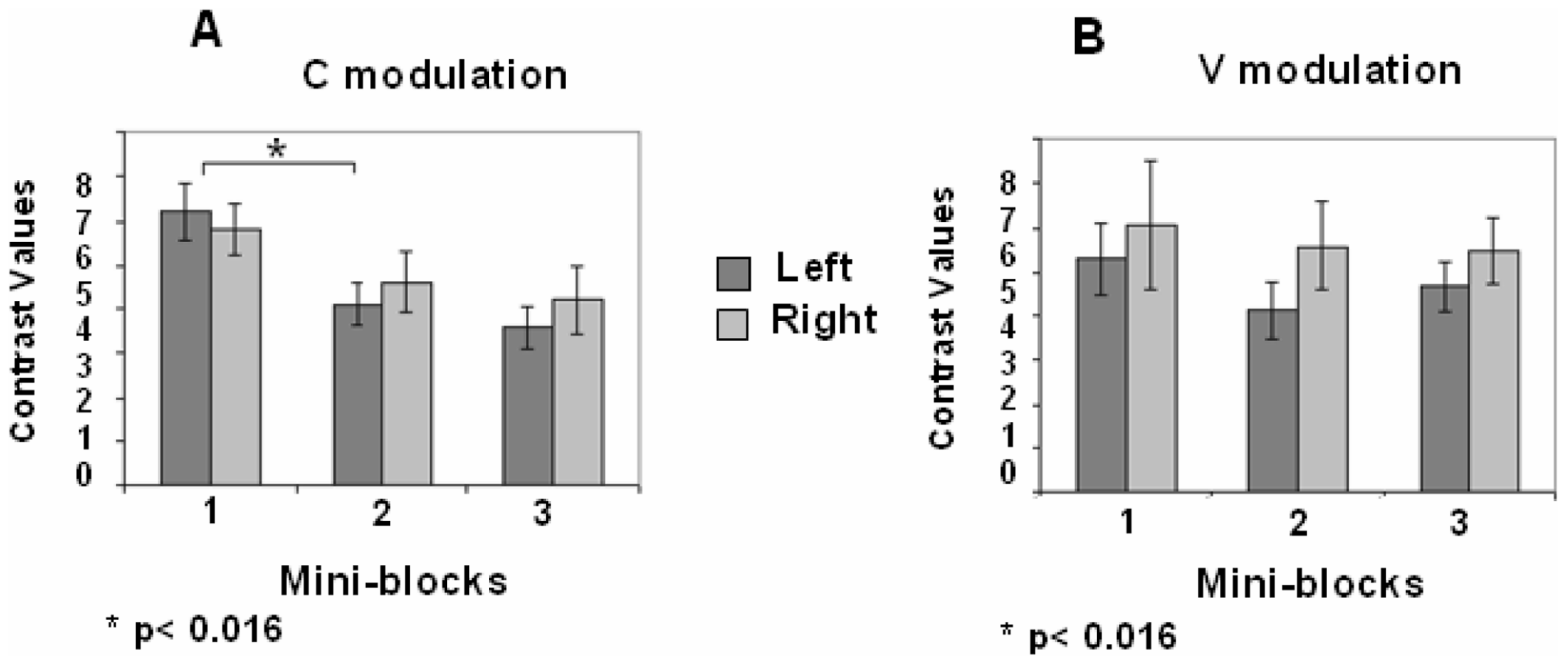
ROI analysis, left and right A1 in the three repeating mini-blocks of the non-semantic task Contrast values (CV) and SEM are shown. (A) during C modulation - single, constant pitch contour for all repetitions of the same word; (B) during V modulation - variable pitch contour between repetitions of the same word. Repetition suppression (RS) effect was found only in the C modulation (*p<0.016, Bonferroni test for multiple comparisons).

Pair-wise comparisons showed significant RS in the C modulation, in the left A1, between mini blocks 1 and 2 (MD = 2.132, SE = 0.46 *p* = 0.001) but not in the V modulation, between any pair of mini-blocks, on either the left or the right A1 (Fig. 9 A and Fig. 9 B).

## Discussion

We were able to show significant repetition suppression (RS) effects in auditory and language related brain areas, during the performance of semantic categorization tasks as well as during passive listening. These statistically significant RS effects, however, were evoked only when each stimulus, a relatively low frequency noun, was repeatedly presented with an invariable prosodic pitch contour (M and P prosodic modulations in the semantic categorization tasks and C prosodic modulation in the non-semantic passive listening task) and not when the target nouns had a different pitch contour on each repeated presentation (V prosodic modulation). These differential RS effects co-occurred with behavioral repetition priming (RP) effects, although the effect of the V modulation on the behavioral measure of RP was somewhat less robust than its abolishing effect on the neuronal phenomenon of RS in auditory and some language processing areas as shown by fMRI. Thus, for words with an invariable prosodic modulation (M or P) there were significant reductions in RT upon the first repetition. However, when the words were presented with a different pitch contour on each repetition (V modulation) RT was only moderately reduced with a significant decrease in RT only between blocks 1 and 3 (i.e., a diminished RP effect).One should note however that RS effects were limited to some but not all activated areas and thus the finding that some behavioral priming survived in the V condition is not surprising. What is surprising and is the most important finding of the current research is that the V modulation of pitch contours significantly reduced the repetition priming effect for the repeated target words and abolished the RS effect in auditory and some language processing areas.

Taken together, our results indicate that implicit memory traces of the pitch contour of a previously heard spoken word facilitated its neuronal processing in auditory and language associated areas, when reencountered, even when the intonation is task-irrelevant; the subsequent recognition and neuronal processing of the same spoken word with an unfamiliar pitch contour (V condition) was clearly less facilitated and the repetition suppression effects were not significant.

### RS in auditory and language related brain areas

Although RP can be robustly demonstrated for repeatedly presented spoken words [39], [40], the co-occurrence of RS has not been consistently demonstrated in auditory cortex or in the neighboring temporal, language related areas. In a number of recent functional neuroimaging studies, in which this issue was addressed [12], [13], [22], [25] no significant priming effects were found in A1 or the STG or MTG. However, recently, Gagnepin et al. [23] reported on RS effects in the right middle/posterior STS and right associative auditory cortex in response to spoken words an acoustically degraded format. Hasson et al. [23] reported statistically significant RS in temporal language related brain areas (right STG and STS, posterior left MTG/STG, bilateral IFG), in response to repetitive exposure to auditory presentations of sentences. In an earlier fMRI study, RS was reported to occur in the right STG and bilaterally in the STS in response to repetitive environmental sounds [21].

Several proposals have been put forward to explain the difficulties in evoking RS in the auditory modality [12], [21]. It was suggested that some tasks, specifically stem completion tasks [13], [22] may be less suitable for demonstrating RS in auditory related brain areas or in temporal language areas [11]. In stem completion tasks, RP is expressed as a tendency to complete, at a faster rate, more word stems to previously heard words relative to words that were not heard before [11]. Bergerbest et al. [21] suggested that functional neuroimaging studies that used stem completion tasks failed to demonstrate RS in auditory related areas because stem completion is more dependent upon phonological representations than on acoustical representations, and according to Schacter et al. [11] and [41] the phonological and acoustical properties of spoken words are represented in separate memory systems. Orfanidou et al. [12] proposed that stem completion tasks provide incomplete perceptual cues for word recognition because they focus on response generation rather than on bottom-up word perception, nevertheless, they were not able to show significant RS in a lexical decision task. No RS was found in a PET study wherein repeated listening to words was used as the task [25] ^.^ Moreover, RS in auditory language related areas was not detected even when behavioral RP was demonstrated [12].

To our knowledge, RS in STG and MTG in response to repeated single spoken words, which were not acoustically degraded, is reported here for the first time. Our current findings are in accord with the findings of Hasson et al. [23] who demonstrated RS in STG and MTG (BA 21 22) in response to repeated spoken sentences. Our participants, however, also showed significant RS in A1 (BA 41 defined anatomically and functionally, by using non-lingual noise) both when using semantic categorization tasks and in passive listening conditions.

One possible explanation for our success in demonstrating RS in auditory and temporal language areas may be related to the use of uncommon (in everyday linguistic environments) pitch modulations for the word stimuli. These comprised of spoken words that were processed and re-synthesized (by PRAAT software) and in which pitch contour was either completely flattened (M modulation) or arbitrarily changed in a manner that is detached from a pragmatic context, i.e., with intonation changes of no task relevant meaning (P and V modulations). RP effects were found to be more robust for uncommon, low linguistic frequency, stimuli [42]. Thus, the re-synthesized, pitch manipulated, words that were used in the current study (both M and P modulation) may have triggered more prominent RS in auditory-language areas compared to the unprocessed (with no artificial pitch modulations) recorded spoken words which were tested in previous functional neuroimaging studies. One should note however that the stimuli used in the current study, were neither unfamiliar (i.e., they were positively recognizable as belonging to the participants native language) nor masked. It is important to draw a distinction between low linguistic frequency stimuli (which we consider the words in the M and P modulations to be) which are associated with enhanced RP behaviorally and RS in language and auditory areas, and unfamiliar or masked stimuli which may result in reduced or no repetition suppression [11], [17]. The re-synthesized words with monotonous or modulated pitch contours used in the current study were lexically familiar and perceptually clear [32], as reflected by the high accuracy of the responses.

In addition to the use of low linguistic frequency stimuli, in the current study a block design paradigm (e.g., [21]) rather than an event related design [12], [22] was employed. RS effects in block design experiments are expected to be stronger than in event-related designs, as was recently demonstrated in the visual cortex [43] because of the intervening stimuli between prime and target that by necessity of design are present in an event related study. Also, the interval between prime and target per-se may be critical [12]. In the current study, in the passive listening task there were actually no intervening stimuli between the prime and tested stimuli. In the two semantic categorization tasks only six words were included in each block (in a different order each time) so that the maximal number of possible intervening stimuli between the first presentation of a given word and its repetition (with the same or different intonation) in the subsequent block was from zero to 10, on average 5 words. In comparison, in the Orfanidou et al. study [12], 12 intervening items were inserted between a given prime and the corresponding target. There was no difference in the time interval between primes and targets (∼30 seconds) in the current study and in Orfanidou et al. study [12].

### Implicit memory for the pitch contour of words of spoken words

Church & Schacter [9] have previously found that minimal changes in the pitch contour of word stems and changes in the intonation of repeated words diminished RP in word recognition tasks. The demonstration of diminished RP by prime-target prosodic pitch variability (pitch manipulation) supported the notion that that “pre-semantic” perceptual auditory attributes of spoken words can be implicitly associated with the words in their memory representation [11]. The results of the current study show that even after a single encounter, memory traces for the pitch contour are established in association with the target word, allowing for substantially more efficient processing if the association is maintained. When the pitch contour stays the same from exposure to exposure (M and P modulations) the spoken word becomes more predictable than when the pitch contour changes upon reoccurrence (V modulation). The lack of RS in the V conditions can be attributed to difficulties in ignoring the changes in the pitch contours in repeated spoken words and thus in specific brain areas the changed pitch contour abolished the “neuronal familiarity” signature even though the very same words (items) were used repeatedly in each of the three blocks of the V condition. Thus, memory traces of the pitch contour of a previously heard spoken word can facilitate its recognition and its processing when reencountered even when the intonation is task-irrelevant, while an unfamiliar pitch contour of the same spoken words does not facilitate its the subsequent recognition. The results lend support for the notion that prosody and specifically pitch contour is strongly associated with the memory representation of spoken words. From this perspective, our findings, that prime-target variation in the pitch contour of words (V modulation) diminished RS in auditory cortex as well as language related brain areas, are in accordance with Schacter's theory [9].

Our results are at apparent odds with previous studies in which a speaker's voice change failed to show significant RP and RS modulations [44], [45]. A possible explanation for the difference between the effect of prosodic variations as effected in the current study and speaker's voice variations on RP and on RS, is that although in both cases an objective physical change is involved, the perceptual system addresses the above two types of physical changes in a different manner. The notion of a “speaker normalization” process, one whereby a given word by different speakers is classified into a single semantic category, is supported by several lines of evidence [7], [45]. The proposal is that the variability related to the different speakers' fundamental frequencies is treated as noise and is equalized, to expose the linguistic message; different speakers' voices need to be disambiguated in order to make the message clear. Prosodic changes, however, consist of pitch contour patterns that may be similarly and commonly represented across different speakers' voices in a given language, with each modulation pattern indicating a differential message. A prosodic change may modulate lexical identification [46], [47] and often entails a change in the meaning of the utterance (intonational meaning) [1]. Thus, a prosodic change may be an important and salient (although sometimes implicit) perceptual auditory cue for auditory-language decoding and may not be stripped at multiple levels of word representation. This would necessitate that unlike different speaker's voices, intonation differences should not be ignored in a bottom-up manner (perhaps at a pre-semantic level, as suggested by Church and Schacter's model [9]). This conjecture may explain why modulation of RS occurred for prime-target changes in the pitch contour of repeated words, while no similar modulation of the RS occurred when the prime-target change was a change in the speaker's voice [12], [13].

The notion that the effect of prime - target prosodic pitch modulation on RP behaviorally, and on RS in auditory and language processing areas, is at least in part a perceptual (pre-semantic) effect is indirectly supported by the results of the non-semantic, passive listening task in our study. In the non-semantic task, participants listened to repeating identical words with either a constant pitch contour (raising or falling) or a changing pitch contour between repetitions (in a manner similar to the V modulation in the semantic task) and were asked only to mark the end of the auditory presentation of each word. Changes in the pitch contour of repeating words diminished RS in auditory cortex (BA 41), bilaterally, as well as in language related areas (BA 41, 21–22).

The changes in the pitch contours, in the current study, were task irrelevant, and moreover, did not affect lexical identity as evidenced by the high levels of performance in both semantic categorization tasks. Nevertheless, one cannot rule out that the reduction of RS in auditory cortex may reflect in part top-down modulation by brain areas engaged in semantic processing. The STG and MTG have been shown to be sensitive to semantics, with reduced activation to spoken target words that were semantically related to prime words [48]. Alternatively, it is possible that the perception of a specific prosodic form (even without semantic implication) may be activated by the prime word, within STG and MTG which are part of a prosody sensitive semantic network [48]. Overlapping units in this network may be activated only when the prime and the target word share an identical prosodic pattern; however, when the intonation is changed between prime presentation and the target words re-introduction, RS may be diminished.

According to a ‘non-traditional’ (connectionist) view of the mental lexicon, words are stimuli whose meaning lies in the causal effects they have on the ‘mental state’ [49]. Thus words are considered as perceptual cues to meaning but do not have canonical meanings, assumed to be stripped of incidental physical-perceptual features according to the traditional concept of the mental lexicon [50], [51]. According to the connectionist view the physical-perceptual aspects of the input may lead to different interpretations by driving (activating) one of a number of mental states as well as by providing specific contextual cues. As prosody is an inherent aspect of experiencing a word (a set of perceptual stimuli) the same word with a different intonation could have a differential effect on the mental state which would be reflected in a different neuronal activation pattern (as shown for example by reduced RS) [16], [17]. The finding of a clear RS modulation effect suggest that even in brain areas engaged in higher level language processing (i.e., beyond perceptual ‘pre-lexical’ stages) the pitch contour of a word is part of it's neural representation even when the prosodic modulation is task-irrelevant.

### Pitch contour modulates RS in IFG

In the semantic categorization tasks, a significant interaction was found between the prosodic modulations and the repetitions of the target words, in the left and in the right IFG (BA 44 45). In the left IFG, significant RS was found in the M and P modulations but not in the V modulation. IFG activation was absent or minimal in the passive listening task.

RS in the IFG was previously demonstrated when participants were required to categorize visually presented words as abstract or concrete [27]. This was interpreted as reflecting conceptual priming, and presumably semantic priming, rather than perceptual non-semantic priming. No RS in the IFG was demonstrated in a perceptual auditory stem completion task [13] RS in the IFG was however described in the context of semantic as well as phonological repetition tasks involving visually presented single words [52] and in studies of semantic priming during lexical decision tasks [53]; but see [48]. Hasson et al. [23] demonstrated RS in IFG in an auditory sentence comprehension task that required judgment but not in a passive listening condition; it was proposed that RS in the IFG may relate to more demanding task conditions. Because no significant activation in the IFG was found in the non-semantic (and less demanding) task (but see, [54]), our findings are equally compatible with the proposal that RS in the IFG is related to more demanding language processing in general or specifically to semantic processing.

Although the prosodic modulations, in the current study, were not specifically designed to bear distinct (linguistic or emotional) intonational meanings, and were task irrelevant, it is possible that the observed priming modulation in left IFG were due to interference by the prosodic modulation on the semantic-conceptual processing of the repeated words. A mode of speech therapy, the modulation intonation therapy (MIT), which is based upon rehearsing exaggerated prosodic pronunciations, was shown in a PET study to reactivate IFG in aphasic patients [55]. One possible explanation for the beneficial effects of MIT, compatible with the current findings, is a facilitation of semantic retrieval processes by prosodic cues.

### Laterality in prosodic processing

Our results showed that the effect of prosodic modulation on RS was as pronounced in STG and MTG in the left as in the right hemisphere. Several recent studies have shown bilateral temporal lobe activation in response to prosodic stimuli. Bilateral activation of the planum temporale was demonstrated in response to auditory sentences with flattened monotonous pitch and to the contour of intonation of sentences per-se (degraded speech) [56]. Bilateral involvement of temporal and frontal lobes in the processing of prosodic modulation of read sentences with different emotional intonation was demonstrated by Kotz and Meyer [57]. Other studies, however, have reported a tendency towards right hemisphere lateralization in response to prosodic modulations [58]. This apparent discrepancy may relate, in part, to acoustic parameters of the stimuli [59]. Pitch modulations occurring over short time intervals, intervals limited to the length of one syllable (i.e., rapidly changing acoustic cues), were reported to activate left temporal areas while pitch modulations over longer time intervals, were reported to activate right temporal areas [59]–[61]. In the latter case, the pitch modulations were expanded to encompass the full length of the words or a sentence (slowly changing acoustic cues). The prosodic modulations applied in the current study combined both relatively rapid changing acoustic cues, at the level of a single syllable, and slower changing acoustic cues, at the level of a whole word. This may account for the bilateral activation in the STG and the MTG, and moreover for the bilateral reversal of RS to the same words when repeated in the V modulation conditions. The M modulation was also characterized by bilateral temporal activation. In this condition, the stimuli, although lacking any of the pitch variability expected in prosodic stimuli, can evoke activity in brain areas engaged in prosodic processing, as these areas are tuned to detect both changes and lack of changes in the pitch contour of spoken words. Thus the M intonation condition may reflect an effort in detecting the missing prosodic pitch cues which may be associated with activation of the same areas that process legitimate prosodic pitch cues. It may be also the case that monotonic speech is processed as a type of prosody which has its own pragmatic and emotional characteristics. Indeed, bilateral activity in STG was reported in an fMRI study of the processing of flattened monotonous speech [56].

Altogether, our results suggest that RS modulation may constitute an effective methodological approach to the investigation of the effects of prosodic modulations of spoken words in the auditory cortex as well as language processing areas. Moreover, our results indicate that implicit memory traces for the pitch contour of spoken words become functional even after a single exposure and are reflected in facilitated neuronal processing in auditory and language associated areas. Thus, pitch contour is strongly associated with the memory representation of spoken words.
